# The triple rule out CT in acute chest pain: a challenge for emergency radiologists?

**DOI:** 10.1007/s10140-021-01911-8

**Published:** 2021-02-18

**Authors:** Vincenzo Russo, Camilla Sportoletti, Giulia Scalas, Domenico Attinà, Francesco Buia, Fabio Niro, Cecilia Modolon, Carlo De Luca, Francesco Monteduro, Luigi Lovato

**Affiliations:** 1 Cardio-Thoracic-Vascular Department, Cardio-Thoracic Radiology Unit, University Hospital S.Orsola-Malpighi, Block 23, Via Massarenti 9, 40138 Bologna, Italy; 2 Emergency Department, Radiology Unit, University Hospital S.Orsola-Malpighi, Via Massarenti 9, 40138 Bologna, Italy

**Keywords:** Coronary CT angiography, CTA, Triple rule out, Chest pain

## Abstract

**Purpose:**

To evaluate the feasibility of triple rule out computed tomography (TRO-CT) in an emergency radiology workflow by comparing the diagnostic performance of cardiovascular and general radiologists in the interpretation of emergency TRO-CT studies in patients with acute and atypical chest pain.

**Methods:**

Between July 2017 and December 2019, 350 adult patients underwent TRO-CT studies for the assessment of atypical chest pain. Three radiologists with different fields and years of expertise (a cardioradiologist—CR, an emergency senior radiologist—SER, and an emergency junior radiologist—JER) retrospectively and independently reviewed all TRO-CT studies, by trans-axial and multiplanar reconstruction only. Concordance rates were then calculated using as reference blinded results from a different senior cardioradiologist, who previously evaluated studies using all available analysis software.

**Results:**

Concordance rate was 100% for acute aortic syndrome (AAS) and pulmonary embolism (PE). About coronary stenosis (CS) for non-obstructive (<50%), CS concordance rates were 97.98%, 90.91%, and 97.18%, respectively, for CR, SER, and JER; for obstructive CS (>50%), concordance rates were respectively 88%, 85.7%, and 71.43%. Moreover, it was globally observed a better performance in the evaluation of last half of examinations compared with the first one.

**Conclusions:**

Our study confirm the feasibility of the TRO-CT even in an Emergency Radiology department that cannot rely on a 24/7 availability of a dedicated skilled cardiovascular radiologist. The “undedicated” radiologists could exclude with good diagnostic accuracy the presence of obstructive stenosis, those with a clinical impact on patient management, without needing time-consuming software and/or reconstructions.

## Introduction

Non-traumatic chest pain is one of the most common complaints in emergency departments (ED), and its evaluation is a very complex issue for ED physicians [1].

Triple rule out computed tomography (TRO-CT) is an electrocardiography (ECG)-gated examination that noninvasively evaluates the coronary circulation and simultaneously visualizes pulmonary arteries and thoracic aorta within a single scan [2]. Because of such capability, TRO-CT stood out as a diagnostic modality in some clinical settings, especially in patients with acute chest pain and low-to-moderate risk for acute coronary syndrome (ACS) in whom pulmonary embolism (PE) or acute aortic syndrome (AAS) should also be considered in the differential diagnosis [3].

TRO-CT can accurately rule out ACS with excellent negative predictive values [4] in the majority of patients with acute chest pain and could identify those with significant coronary artery stenosis. Moreover, anatomic imaging of the whole chest with TRO-CT can also detect non-coronary causes of acute chest pain, helping emergency department physicians to rapidly direct patients to the most appropriate in- or out-patient treatment [5].

In a selected population, TRO-CT is able to provide a cost-effective evaluation reducing diagnostic time [6], with lower costs and fewer repeat evaluations for recurrent chest pain, as compared with standard of care [7, 8]. At the same time, TRO-CT requires time-consuming imaging interpretation by subspecialist cardiovascular radiologists and it is still controversial if their assistance is required to perform TRO-CT in an emergency setting [9].

Therefore, the purpose of our study was to evaluate the feasibility of TRO-CT in an emergency radiology setting comparing the diagnostic performance of cardiovascular and general radiologists in the interpretation of TRO-CT studies in patients with acute and atypical chest pain.

## Material and methods

### Study population

Between July 2017 and December 2019, 350 adult patients (203 men and 147 women, mean age 62.9 ± 17.6 years, range 20–93) underwent TRO-CT studies for the assessment of atypical chest pain. Population characteristics are summarized in Table 1.

**Table 1:** Population characteristics

Sex	
- Men	203 (58%)
- Women	147 (42%)
Mean age	62.9 ± 17.6 years
Mean BMI	27 ± 6.4 kg/m
Cardiovascular risk factors	- Hypertension 200 (57%)
	- Hypercholesterolemia 140 (40%)
	- Obesity 74 (21%)
	- Smoking 122 (35%)
	- Diabetes 42 (12)
	- Heredity 56 (16%)

Patients were eligible for TRO-CT only if they had a low-to-intermediate cardiac risk for acute coronary syndrome (negative cardiac bio-markers and non-diagnostic ECG), and if the presence of acute PE or AAS could not be clinically excluded.

Exclusion criteria were adverse reaction to iodinated contrast agents, traumatic chest pain, and high risk for ACS.

### Scanning protocols

In the absence of contraindications, to achieve maximum coronary vasodilatation, sublingual nitroglycerin (5 mg) was administered 2–3 min before the start of TRO-CT and intravenous β-blocker (metoprolol 5–15 mg) was used in patients with heart rates > 65 beats/min, in order to reduce heart frequency (and also extrasystoles) and improve image quality. Blood pressure and heart rate were monitored before, during, and after the administration of nitroglycerin and β-blocker.

All TRO-CT examinations were acquired with a 128-slice CT scanner (Brilliance iCT SP, Philips, The Netherlands).

Primarily, a non-contrast scan of the entire chest was acquired in order to evaluate aortic and lung abnormalities, as well as severe coronary calcifications.

After that, contrast media (95 ml of Iomeron 400, Bracco, Italy) was injected through a 16–18 gauge intravenous catheter placed into a large antecubital vein using a biphasic “Dual Flow” injection protocol with a double syringe injector (Stellant Flex with Certegra workstation, Medrad, Warrendale, PA, USA): 70 mL of contrast media followed by a simultaneous bolus of 25 mL of contrast media and 25 mL of saline, injected at a flow rate of 5.0 mL/s. With bolus tracking technique, the CT scan (ECG-gated acquisition from the level of the lung apices through the diaphragm) started automatically 5 s after a threshold of 200 UH was reached in the left atrium.

Depending on patient’s heart rate, rhythm and age, prospectively ECG-triggered or retrospectively ECG-gated acquisitions were used.

For exam quality assessment, a 5-point scale was used as follows: 1 = Poor, nondiagnostic quality due to severe artifacts; 2 = Sufficient quality, with significant blurring or stair-step artifacts but diagnostic quality; 3 = Average quality, with some blurring or stair-step artifacts not affecting significantly the image assessment; 4 = Good quality, with minor blurring artifacts; 5 = Excellent quality, with no artifacts.

Image quality assessment was checked and performed by the cardioradiologist who executed and reported the examination. All examination with poor quality (5 more patients) were so previously excluded from the study as, thus, from further evaluations.

### Image assessment

Three radiologists with different expertise retrospectively and independently reviewed the studies:

- a “dedicated” radiologist with 10 years’ experience in cardiovascular imaging (CR);
- a “general” radiologist with 25 years’ experience in emergency radiology (SER);
- a “general” radiologist with 5 years’ experience in emergency radiology (JER).

**Fig. 1:**
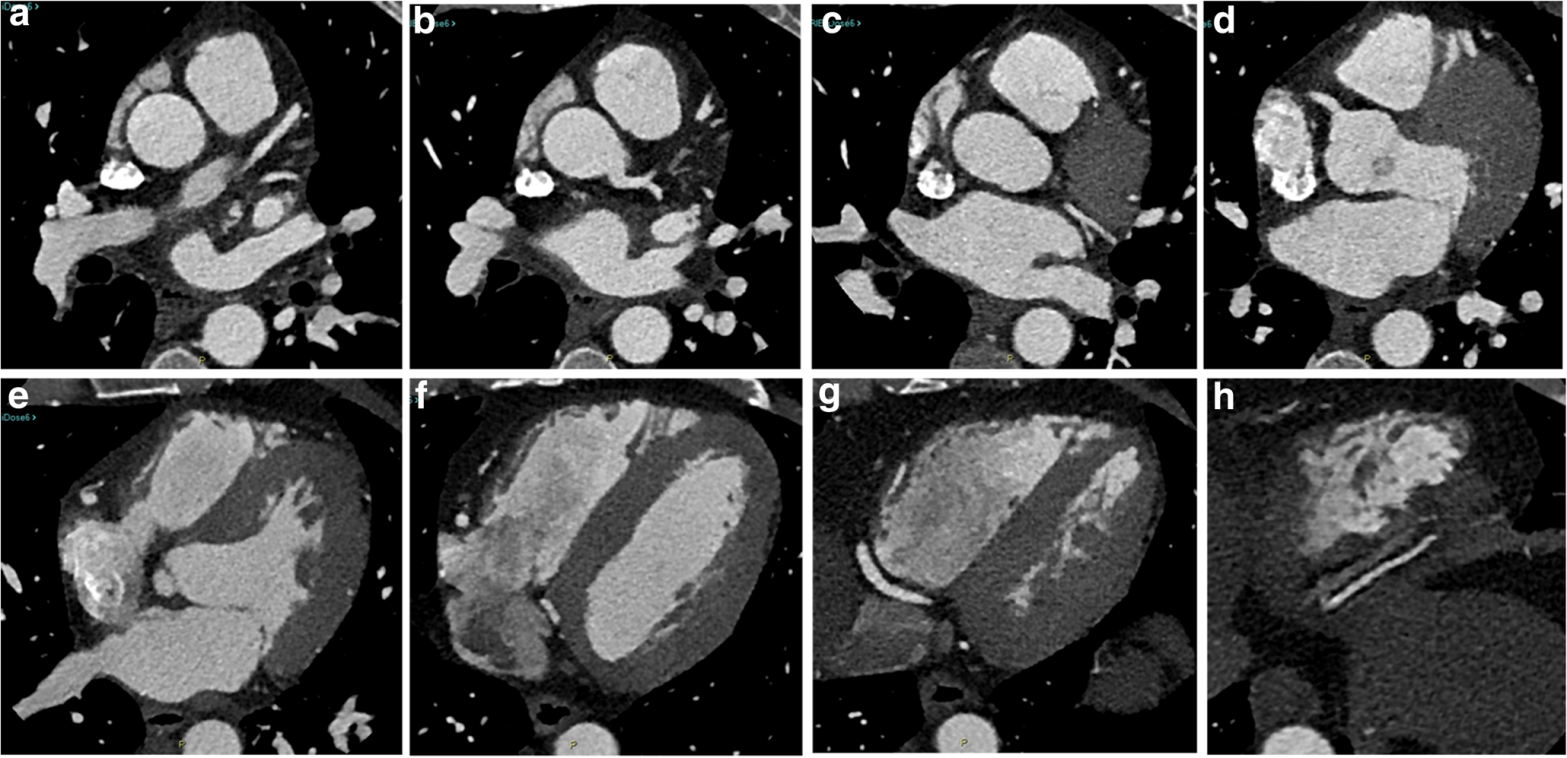
An example of axial TRO-CT images (**a**–**h**) used for coronary arteries post-processing

**Fig. 2:**
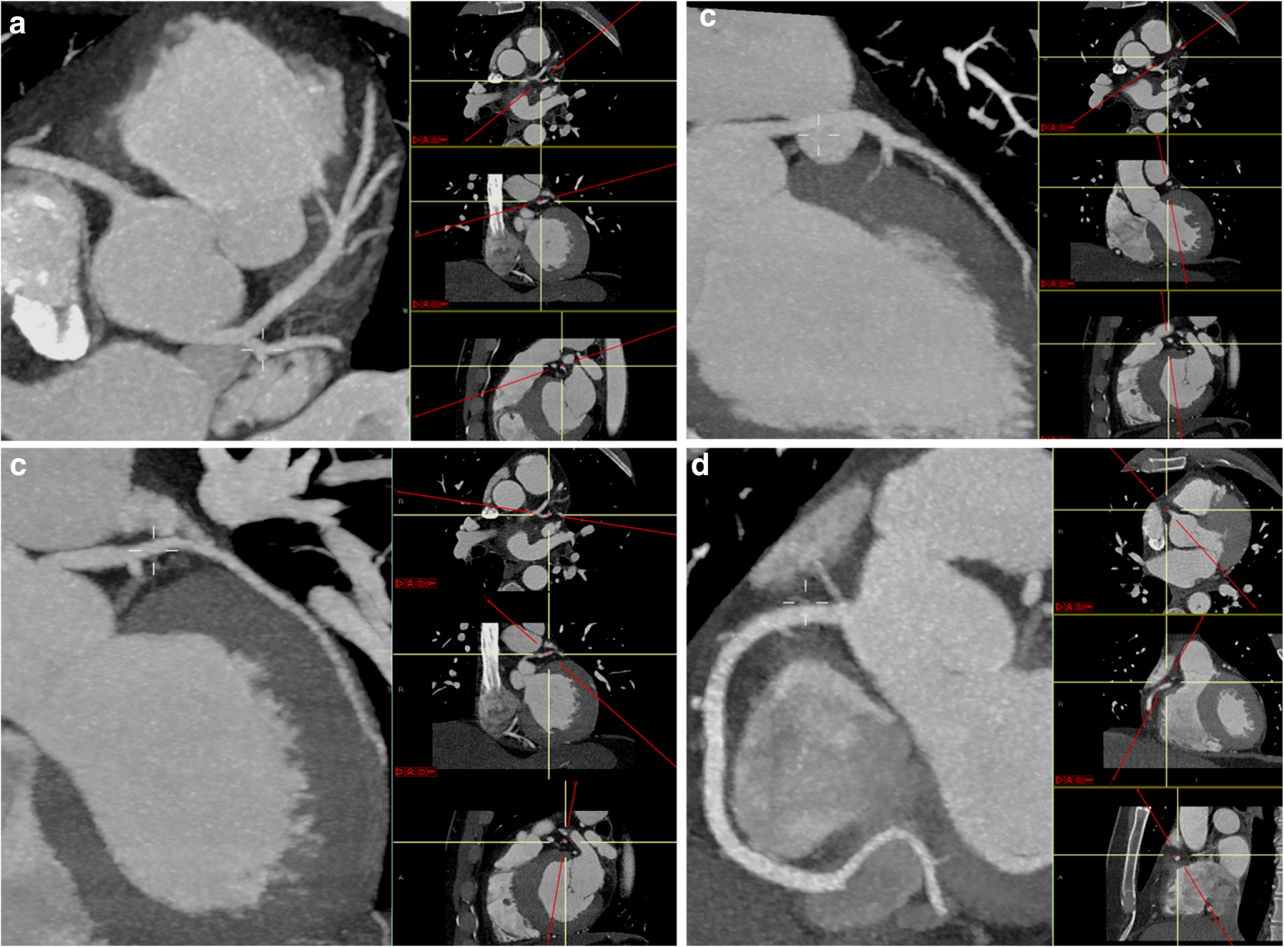
MPR-MIP reformatted images for right (**a**, **d**) and left (**a**, **b**, **c**) coronary artery evaluation. On the right portion of each image, the orientation along the three major anatomic planes (axial, coronal, and sagittal) is clearly showed (red lines)

**Fig. 3:**
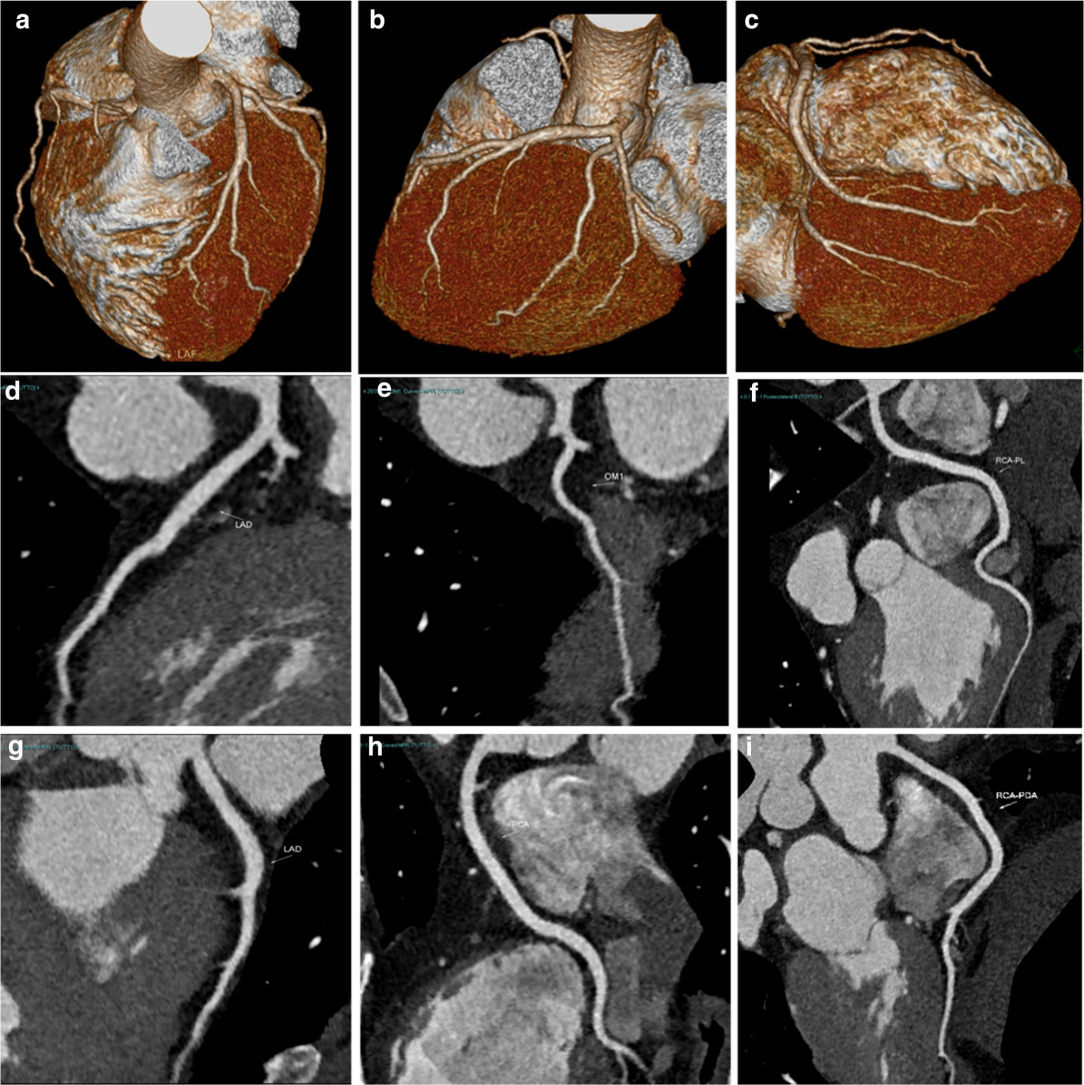
Volume rendering (**a**–**c**) and curved-MPR (**d**–**i**) images, normally used for coronary artery evaluation on dedicated workstations

Studies were reviewed on standard workstations using just axial reconstructions (Fig. 1) and multiplanar (MPR) projections (Fig. 2). No thin slab, curved-MPR, volume rendering, or more advanced post-processing software (such as vessel analysis or coronary extraction) were applied, mainly for time-saving reasons in an emergency setting (Fig. 3).

In case of retrospectively gated scan, best cardiac phases were selected from those available, generally 75% of R-R interval for heart rates < 70–75 bpm and 35–45% for higher heart rates.

The revision of all TRO-CT studies was performed by each one of the three radiologists, who evaluated the presence/absence of PE, AAS, or coronary artery disease (CAD). For evaluation of CAD, any stenosis greater than or equal to 50% was considered as obstructive. Each major coronary artery (left main, left anterior descending, left circumflex, and right coronary artery) and all significant obtuse marginal and diagonal branches were evaluated for stenosis, using qualitative measurements: categorization of luminal narrowing was based only on subjective visual assessment, in all cases.

For the assessment of diagnostic accuracy of all revised examinations, the original report of the TRO-CT study by an experienced cardio-radiologist was considered as reference standard.

All patients gave informed consent to the study, approved by our institutional review board as retrospective analysis of routinely performed CT scans.

Statistical analysis was performed with SAS® University Edition: distribution analysis to evaluate and understand the characteristics of the different populations, confusion matrix (*k*) to understand performance of matching /non matching patients, correlation analysis between variables, and logistics univariate and multivariate analysis to verify the null hypothesis that the two populations behave similarly. The null hypothesis was verified by performing a Student’s *t* test with *p*<0.05. A low probability, as obtained, determines the likelihood that there are in fact variations between the samples.

## Results

Among 350 TRO-CT scans, 310 (88.7%) were performed with retrospective ECG-gating with an average dose of 11.79 mSv (mean heart rate = 63 bpm), while the remaining 40 TRO-CT (11.3% of patients) were acquired with prospective ECG-triggering with an average dose of 6.13 mSv (mean heart rate = 54 bpm).

Patients with no evidence of coronary atherosclerosis or with minimal-to-mild (0–49%) CAD were considered as “negative” for obstructive CAD, whereas patients with moderate CAD to completely occluded coronary artery (50–100%) were classified as “positive” for obstructive CAD.

The image quality was excellent in 121 pts (34.58%), good in 137 pts (39.11%), average in 40 pts (11.44%), and sufficient in 59 pts (16.87%).

All *p* values for the abovementioned null hypothesis were statistically significant (*p*<0.05) and, consequently, not explainable by the randomness of the sampling.

All physicians have properly recognized PE and AAS with a concordance rate of 100% (*k* =1)

About CAD, the comparison with reports made by an expert cardiovascular radiologist with the help of all available software for coronary analysis showed the following global concordance rates: 94.51% (*k* = 0.71), 89.9% (*k*= 0.65), and 89.73% (*k* = 0.62), respectively, for CR, SER, and JER.

**Fig. 4:**
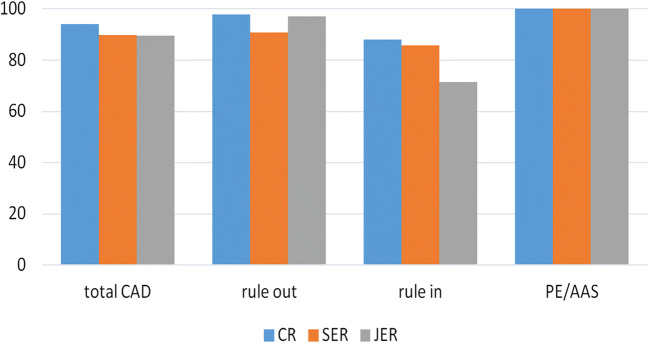
Concordance rate of diagnosis of PE, AAS, and total CAD between a senior cardioradiologist (as “gold standard”), who previously evaluated studies using all available analysis software, and another cardioradiologist, a senior emergency radiologist, and a junior emergency radiologist who evaluated studies only by MPR analysis. CAD is considered as non-obstructive CAD (rule out) and obstructive CAD (rule in)

Moreover, the concordance was also calculated in patients with and without obstructive CAD. In particular, the concordance rates in excluding obstructive CAD (“rule out”) for CR, SER, and JER were, respectively, 97.98%, 90.91%, and 97.18%. “Ruling in” obstructive CAD was more challenging: concordance rates were 88.32% (*k* = 0.57) for CR, 85.9% (*k* = 0,54) for SER, and 71.57% (*k* = 0.42) for JER. Results are summarized in Fig. 4.

Finally, considering the two non cardiovascular radiologist (SER and JER), a sort of “learning curve” was also evaluated analyzing concordance rates in the first and in the second half of reviewed cases: in the first 175 patients, global concordance rates were 85.7% for JER and 88.3% for SER, while in the last 175 patients concordance rates were respectively 93.7% and 90.9%.

## Discussion

Evaluation of chest pain and possible ACS is a challenging task for emergency departments.

A large number of studies, including meta-analysis, have demonstrated a high negative predictive value for coronary CT angiography (CCTA) and TRO-CT for the exclusion of significant CAD [10]. Thus, such noninvasive examinations are prone to be increasingly important for emergency departments in the context of clinical risk stratification of patients with chest pain and suspected ACS: patients without significant CAD, AAS, or PE could be quickly discharged, while others must be hospitalized and those with moderate-to-severe CAD at coronary CT should be scheduled for further evaluation [11].

Cardiac CT imaging requires competence on many levels [12]. Image reconstruction and post-processing need knowledge in CT physics, radiology, and cardiac physiology [13]. Finally, skills on image interpretation should be based on knowledge and experience in CCTA, as well as detailed knowledge of cardiac anatomy, normal and variant patterns of the coronary circulation, and a thorough clinical background in CAD assessment.

Because of the complexity of coronary anatomy [14], the frequency of motion and calcium-related image artifacts [15], and the morphologic subtleties of lesions, interpreters must review CCTA interactively on cardiac-specific interpretation software platforms capable of bi- and tri-dimensional displays in all conventional reconstruction formats. These include trans-axial bi-dimensional image stacks, multiplanar reformations (MPR), maximum intensity projections (MIP), curved multiplanar reformations (cMPR), and volume rendering technique (VRT) reconstructions [16]. Furthermore, quantification of the luminal stenosis (diameter and area) and plaque analysis (composition, extent, and morphology), which are available using specific tools, may assist image interpretation; studies have reported that CCTA quantification of lesion severity (in particular percentage of diameter stenosis) has a good general correlation with quantitative invasive angiography and intravascular ultrasound, but with a relatively large standard deviation.

For these reasons, we sought to evaluate the feasibility of TRO-CT in an emergency radiology setting, comparing the diagnostic performance of cardiovascular and general radiologists in the interpretation of TRO-CT studies in patients with acute and atypical chest pain.

The retrospective review of our 350 cases by three operators with different experience in the field, using just axial and MPR images for post-processing, showed approximately 90% of global concordance rate (*k* = 0.62-0.65) for emergency—noncardiovascular—radiologists (SER and JER), and almost 95% (*k* = 0.71) for the cardiovascular radiologist. The concordance rate was higher in ruling out significant CAD (up to 97% for JER).

Therefore, our data are consistent with the results of a recent study concerning the residents’ performance in the interpretation of TRO-CT and confirm the feasibility of TRO-CT studies in the regular workflow of radiologists in an emergency department: ruling out obstructive CAD is absolutely realistic for an emergency radiologist (91–97% concordance rate), who could have almost the same accuracy of a cardiovascular radiologist, if properly trained (together with radiology technicians) about technical aspects of the CCTA acquisition [17].

**Fig. 5:**
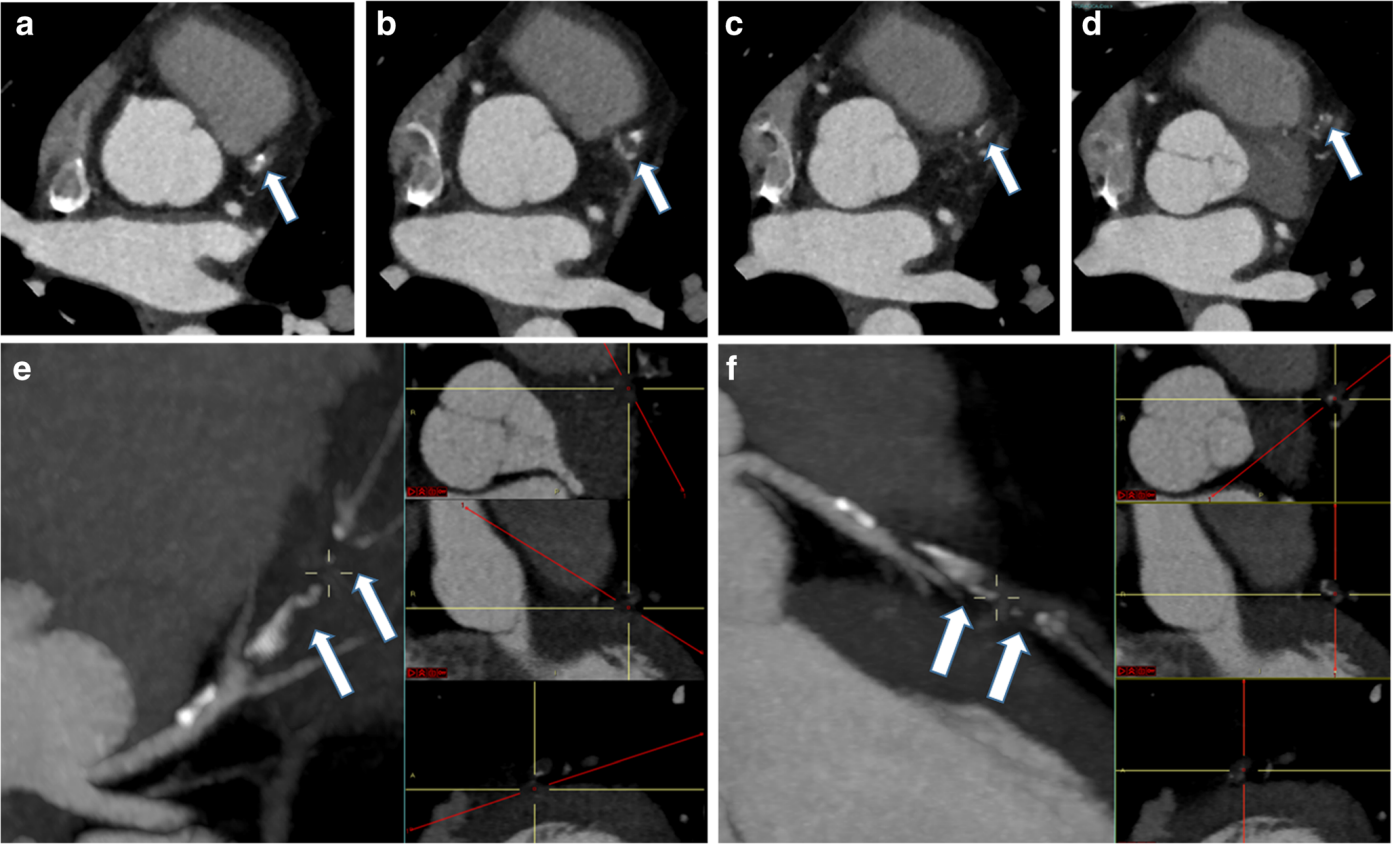
Axial (**a**–**d**) and MPR-MIP (**e**, **f**) CT images from a patient with obstructive CAD on left anterior coronary artery (arrows). As in Fig. 2, on the right portion of **e**–**f** images, the orientation along the three major anatomic planes (axial, coronal, and sagittal) is also showed (red lines)

**Fig. 6:**
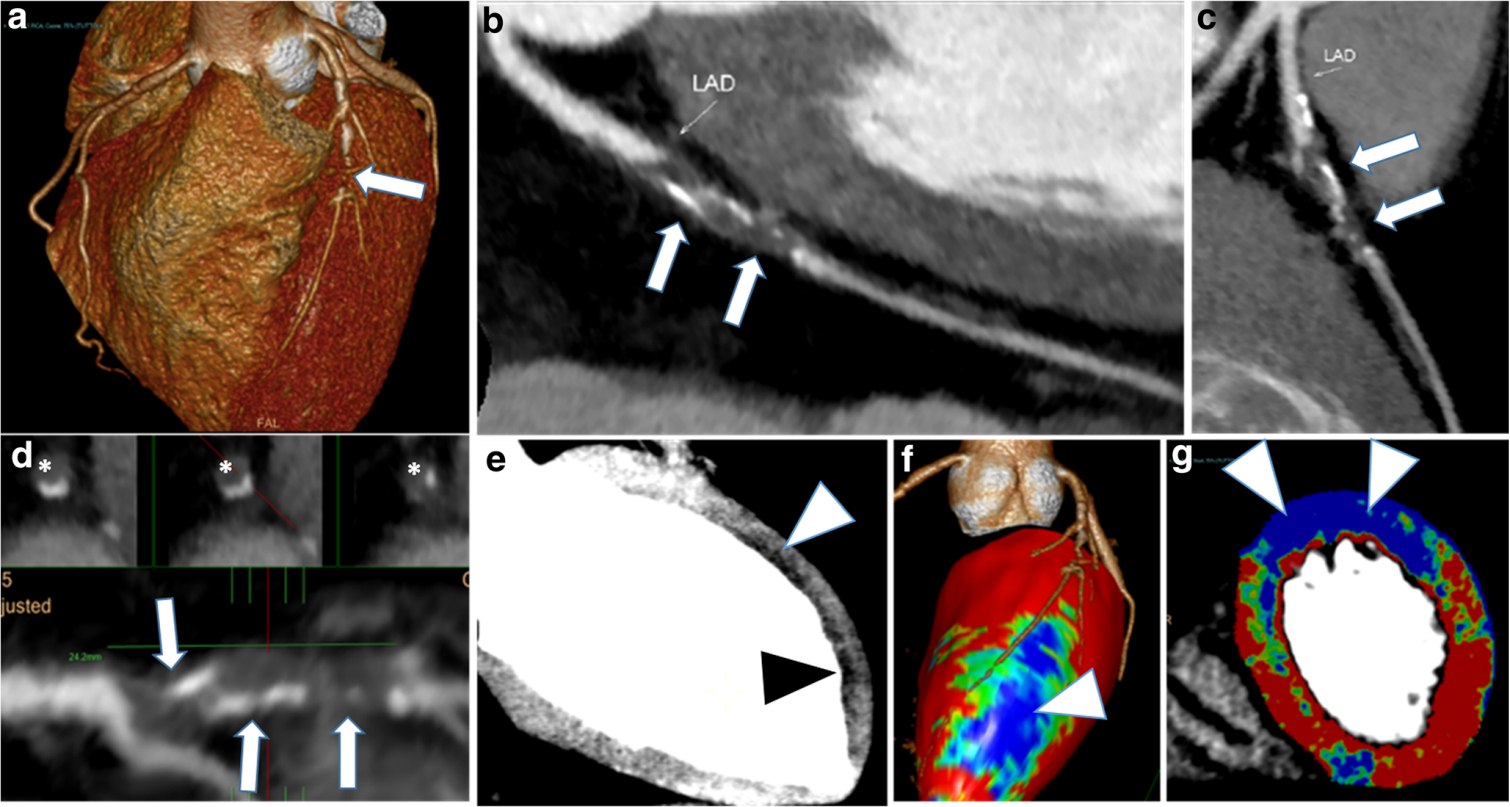
The same case of Fig. 5, previously post-processed using all available dedicated software: volume rendering (**a**), curved-MPR (**b**, **c**), vessel analysis with straight and cross-section views (**d**), MPR thick (**e**), and perfusion defect analysis (**f**, **g**). The obstruction of left anterior descending coronary artery is showed by arrows (**a**–**d**) or asterisks (cross-sectional images in **d**), while subendocardial perfusion defects along the anterior wall are evidenced by arrowheads (e–**g**)

Consequently, in an emergency setting, TRO-CT is not a so “special” examination reserved for highly trained subspecialists only, and it is also not so time-consuming when using just basic image post-processing, even with a drawback of a roughly 6% (3–9%) of concordance (and, thus, accuracy) loss in ruling out obstructive CAD when compared with an expert cardiovascular radiologist who also used all available and time-consuming post-processing software (Figs. 5 and 6).

Curiously, emergency radiologists showed two different trends: a tendency to underestimate the positive patients for the radiologist with less experience (JER) and to overestimate the negative patients for the radiologist with greater seniority (SER).

Finally, the observed variation of correlation percentages among emergency radiologists between the evaluation of the first and second half of patients (in chronological order), with a progressive increase of agreement, could be attributed to training. After the review of more than 150 cases, emergency radiologists got expertise becoming “skilled” in choosing the best cardiac phase for images reconstruction and about grading the severity of CAD.

In our study, we exclusively considered the concordance rate between the evaluation of TRO-CT in an emergency setting, using only axial and MPR reconstructions without other time-consuming post-processing software, and the evaluation of TRO-CT in a cardiovascular setting, using dedicated post-processing software.

### Study limitations

The major limitation, not surmountable due to the lack of conventional coronary angiography in most of the patients (in particular in all of patients with a negative CT scan), was the use of the original report made by a senior cardioradiologist as the reference. This atypical “gold standard” could not be 100% accurate, especially for significant stenoses: as reported in literature [18], CT has a very high negative predictive value (mean value of 99%, range 98–100%), but a lower positive predictive value (mean value of 93%, range 89–98%). This latter (presence of few false positives) could have generated some errors among readers (CR, SER, and JER).

Another limitation is that cardiac CT examinations were not performed by emergency radiologists but by cardioradiologists and their technicians. So emergency radiologists were not involved in the acquisition phase, which is not of immediate management (scanning and injection protocols, drugs, and more). This could be overcome by a tailored training of emergency radiologists about technical aspects of CCTA acquisition and images assessment, which is mandatory in order to improve the accuracy.

Finally, due to the nature of the study (feasibility), we did not assess the diagnostic accuracy of TRO-CT comparing the patients’ outcome.

## Conclusion

These preliminary data lead us to consider the feasibility of the TRO-CT also in an emergency radiology department that cannot rely on the full 24/7 availability of a cardioradiologist. In our experience, a non-cardiovascular radiologist can rule out with good accuracy (up to 97%) the presence of obstructive CAD—the one with a clinical impact on patient management—without the need of time-consuming reconstructions.
